# Inhibiting an inhibitor: a decoy to recover dexterity after spinal cord injury

**DOI:** 10.1093/brain/awaa175

**Published:** 2020-06-15

**Authors:** Elizabeth J Bradbury, Raquel Oliveira

**Affiliations:** King’s College London, Regeneration Group, Wolfson Centre for Age-Related Diseases, Institute of Psychiatry, Psychology and Neuroscience, Guy’s Campus, London, UK

## Abstract

This scientific commentary refers to ‘Nogo receptor decoy promotes recovery and corticospinal growth in non-human primate spinal cord injury’, by Wang *et al.* (doi:10.1093/brain/awaa116).

**This scientific commentary refers to ‘Nogo receptor decoy promotes recovery and corticospinal growth in non-human primate spinal cord injury’, by Wang *et al.* (doi:10.1093/brain/awaa116).**

Worldwide, an estimated 27 million people are living with the effects of a traumatic spinal cord injury, with >250 000 new injuries suffered each year (GBD 2016 Traumatic Brain Injury and Spinal Cord Injury Collaborators, 2019). Healthcare costs are among the highest of any medical condition, ranging from GBP 0.47–1.87 million per individual over their lifetime, with tetraplegia incurring the highest costs (McDaid *et al.*, 2019). Personal costs to individuals facing a lifetime of dependence and disability are incalculable. Along with loss of sensory function and paralysis, many patients suffer incontinence, chronic pain and depression. Most spinal cord injuries occur in the neck (cervical) region (https://www.nscisc.uab.edu/) and cause disability in the upper limbs and hands. Losing the ability to reach, grip, hold and pick up objects can severely limit independence and quality of life. Current treatment options are mainly limited to early surgical intervention for mechanical decompression, symptomatic relief, supportive care and rehabilitation. New therapies are urgently needed. A number of promising regenerative therapies are currently being explored in preclinical studies (recently reviewed in Hutson and Di Giovanni, 2019). These broadly encompass two main approaches: (i) strategies to target the ‘poor intrinsic capacity’ for neural repair, for example by modulating the genetic and transcriptional profile of injured neurons, neural stem cell transplantation and modulation of neuronal activity; and (ii) strategies to target the ‘extrinsic inhibitory environment’ of the injured spinal cord, for example by blocking or neutralizing growth inhibitors that are highly expressed after injury and that play a role in restricting neuronal growth and neuroplasticity. In this issue of *Brain*, Wang and co-workers take the second approach of ‘inhibiting an inhibitor’ and describe a series of preclinical safety and efficacy studies in rodents and non-human primates to test the potential of a Nogo receptor decoy as a treatment for spinal cord injury (Wang *et al.*, 2020).

Two major classes of neuronal growth inhibitors are abundantly expressed after traumatic spinal cord injuries, those associated with tissue scarring and gliosis (Bradbury and Burnside, 2019) and those associated with myelin (Schwab and Strittmatter, 2014). Myelin-associated inhibitors have been a target for regenerative therapies for over 30 years, since Martin Schwab’s group first identified a potent neurite growth inhibitor associated with oligodendrocytes and myelin fractions, later identified as Nogo-A. Decades of research have subsequently led to the development of numerous strategies to block or inhibit this inhibitor, with robust demonstrations of enhanced neuroplasticity of motor pathways associated with improvements in limb mobility, locomotion and upper limb function in models of spinal cord injury and stroke (reviewed in Schwab and Strittmatter, 2014). Of these, antibodies that block Nogo-A function have been widely applied in rodent and non-human primate models of spinal cord injury and recently in humans (Sartori *et al*., 2020). Another strategy to prevent Nogo-A’s inhibitory actions is to block its signalling by targeting the Nogo-66 receptor 1 (NgR1). Targeting NgR1 is a particularly potent approach, as other myelin-associated inhibitors implicated in growth cone collapse and inhibition of neurite outgrowth also bind and signal via this receptor, including myelin-associated glycoprotein and oligodendrocyte myelin glycoprotein. AXER-204 is a recently developed soluble human fusion protein that acts as a decoy, or trap, for these myelin-associated growth inhibitors, preventing their signalling and promoting neuronal growth. Having previously tested this Nogo receptor ‘decoy’ protein in rat contusion injury models (Wang *et al.*, 2006), in this latest work the authors use non-human primates with cervical level injuries to study toxicological, behavioural and neurobiological effects of AXER-204. The results reveal no observable toxicity in rats or primates, increased regenerative growth of a major descending motor pathway, and recovery of forelimb use in monkeys (Fig. 1).

**Figure 1:**
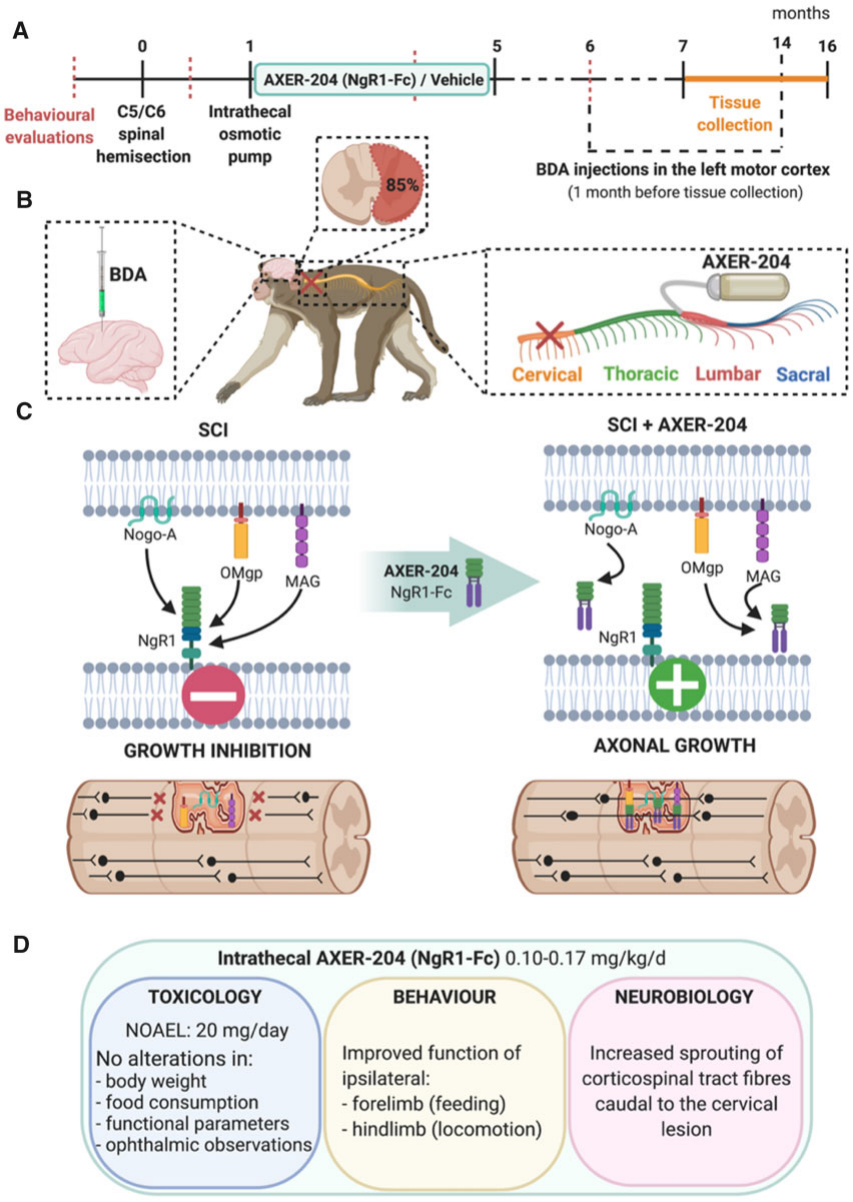
**Schematic of experimental design and key findings.** (**A**) Timeline of the experimental protocol showing time points of behavioural evaluation, spinal cord hemisection injury, delivery of AXER-204 (NgR1-Fc) or vehicle over 4 months, biotinylated dextran amine (BDA) tracer injections and tissue collection between 7 and 16 months after injury. (**B**) Schematic representation of surgical protocols performed in African green monkeys, depicting the unilateral hemisection injury at cervical level C5/C6, intrathecal catheter implantation at the lumbar level for continuous infusion of the drug via a connected minipump and BDA injections into the left motor cortex to label descending axons of the corticospinal tract. (**C**) Illustration of molecular events occurring after spinal cord injury and in response to treatment with AXER-204. Following spinal cord injury (SCI), myelin-associated neuronal growth inhibitors such as Nogo-A, myelin-associated glycoprotein (MAG) and oligodendrocyte myelin glycoprotein (OMgp) are intensely expressed and bind to the Nogo-66 receptor 1 (NgR1), causing growth cone collapse and inhibiting neurite outgrowth. Intrathecal treatment with AXER-204, the Nogo receptor decoy, traps these myelin-associated growth inhibitors, effectively blocking NgR1 signalling, which enables axonal growth and neuroplasticity to occur within the normally inhibitory spinal cord injury environment. (**D**) AXER-204 delivered intrathecally to non-human primates with cervical level spinal cord injuries has a favourable toxicology profile, promotes recovery of forelimb function during feeding and hindlimb locomotor function in the open field, and enables regeneration of the corticospinal tract, a major descending motor pathway important for skilled voluntary control. NOAEL = no observed adverse effect level. Image created with BioRender.com.

First, dose escalation and toxicity studies were carried out in both rodents and non-human primates, including chronic intrathecal and intravenous administration in rats (over 2–4 months) and chronic intrathecal administration in monkeys (over 3.5 months), at doses far greater than would be applied in humans. Numerous measures of toxicity and clinical observations (including body weight, food consumption, electrocardiographic measurements, respiration rate and ophthalmic observations) revealed no toxicity or adverse events related to AXER-204, suggesting a good safety profile. Pain sensitivity was not specifically tested, although animals were scored on a neurological scale that includes a sensation response and no differences were observed between AXER-204 and vehicle-treated groups. However, it is important to note that aberrant sprouting and abnormal sensitivity to innocuous or painful stimuli is one potential negative outcome of unblocking neuronal growth inhibitors, particularly with agents that promote neuroplasticity. Inclusion of pain sensitivity testing may therefore be an important consideration for future clinical trial design.

Long-term efficacy studies were then carried out in non-human primates. The study was well powered, particularly for a primate study, and well-designed. A total of 13 primates across two cohorts completed the full study (*n *=* *7 with AXER-204; *n *=* *6 with vehicle), with a randomized treatment design and researchers blinded to treatment group at each stage (including surgeons, animal handlers, behavioural scorers and histologists). African green monkeys received a lateral hemisection injury (a complete cut through the right side of the spinal cord) at the cervical (C5/C6) level. One month after injury, the monkeys were fitted with minipumps that enable continuous controlled drug infusion, placed under the skin between the monkey’s shoulder blades and connected to a catheter with the tip secured intrathecally at the lumbar spinal level. AXER-204 (or vehicle) was infused into the spinal cord over 4 months, with pumps replaced once a month (Fig. 1A and B). Hand usage during feeding and hindlimb function in the open field were evaluated by analysing video-recorded observations prior to injury, and at three post-injury time points (before treatment, in the fourth month of treatment and 1 month after treatment cessation; Fig. 1A). Forelimb preferences were calculated as the number of times animals attempted to use the right hand or both hands to retrieve food from the top of the cages. Hindlimb activity was measured by joint movements, weight bearing, and digit function observed while grasping cage bars. Prior to injury, monkeys used right and left forelimbs equally for feeding, while injury led to disuse of the affected right forelimb. Monkeys treated with AXER-204 showed an increase in right forelimb usage and a decline in left-side preference over time. Hindlimb function was also significantly improved after AXER-204 treatment, in measures of joint movement, weight bearing and digit usage. Note, some additional behavioural time points might have provided a more complete understanding of the time course of recovery. For example, determining at what point in the treatment regimen recovery began, whether recovery continued over the treatment period or whether (and when) it reached a plateau and, importantly, whether recovery was maintained over long-term chronic post-injury time points. Monkeys remained in the study for up to 16 months post-injury, but the last behavioural assessment was carried out at 6 months. Some information on skill and dexterity while handling, holding and grasping food, in addition to hand use preference, would also have been informative. Nevertheless, the observed recovery was impressive, and the fact that it was still evident a full month after cessation of drug treatment suggests that long-term neural rewiring may have occurred and highlights the relevance of this approach for treating chronic spinal cord injury.

Finally, neurobiological assessments were performed in spinal cord tissue sections obtained 7–14 months after injury. The completeness of the lesion was examined and a similar extent of injury (85% complete hemisection) was observed in both treatment groups (Fig. 1B). The authors also evaluated several markers of gliosis and inflammation and saw no differences in tissue scarring, matrix deposition or inflammatory cell infiltration. Thus, the observed behavioural recovery in AXER-204 treated monkeys cannot be attributed to lesion variability or tissue sparing and is more likely due to new connectivity of motor pathways. The authors explored this possibility by examining regenerative growth of descending axonal pathways. No changes were observed in descending serotonergic axonal projections. However, corticospinal tract labelling (using neuroanatomical tracer injections in the primate motor cortex; Fig. 1B) revealed abundant axonal projections above the injury in both groups but significantly increased axon density below injury only in animals treated with AXER-204. Similar increases in corticospinal axon densities below the lesion in AXER-204 treated monkeys were observed at both time points studied (6–7 or 12–14 months post-injury), indicating that new connectivity was maintained even at long-term chronic stages, over 6 months after cessation of treatment.

This study is of high clinical relevance, given the focus on cervical level injuries (the most common location of human spinal cord injuries), the observed recovery in hand function (one of the highest rated priorities for individuals living with spinal injuries) (Anderson, 2004), and the application of AXER-204 at a chronic post-injury time point (indicating its relevance to the majority of individuals currently living with long-established injuries). The findings in primates, in addition to the solid basis of experimental studies in rats and the favourable toxicity profile clearly support the clinical progression of AXER-204. Indeed, a clinical trial for AXER-204 in participants with chronic spinal cord injury is currently recruiting (ClinicalTrials.gov Identifier: NCT03989440). It remains to be seen whether the recovery observed with AXER-204 treatment would be further enhanced if combined with an additional therapy (Griffin and Bradke, 2020), for example strategies to neutralize scar-associated inhibitors (Bradbury and Burnside, 2019), or other methods to boost regenerative capacity (Hutson and Di Giovanni, 2019). Certainly, it is expected that AXER-204 would be combined with a programme of rehabilitative training, since this is routinely applied in the clinic. It will be interesting to see the extent to which such training will harness the neuroplasticity potential of AXER-204, perhaps by shaping and strengthening useful connections.

With the burgeoning advances in our knowledge of what limits tissue repair, regeneration and neuroplasticity after spinal cord injury, the advanced preclinical stages of several promising therapeutics, and a number of ongoing and planned clinical trials, this is a hopeful time for experimental regenerative therapies to become realized as clinical treatments. We await the results of clinical trials with AXER-204 with great anticipation and expect that this will soon be one of a range of neuroplasticity-promoting therapies to become available in the clinic. With these treatments, the possibility of restoring functions such as upper limb mobility and hand dexterity to those with paralysing injuries is drawing ever closer.

**Glossary**

**AXER-204 (also known as Nogo receptor decoy; NgR1-Fc, AXER-204; Nogo Trap):** A soluble human fusion protein that acts as a decoy/trap for multiple myelin-associated neuronal growth inhibitors including Nogo-A, myelin-associated glycoprotein and oligodendrocyte myelin glycoprotein.

**Corticospinal tract:** A major descending motor pathway important for skilled voluntary control, including fine control of hand and finger movements.

**NgR1 (Nogo-66 receptor 1):** A receptor that when activated signals growth inhibition. It has multiple ligands, including the Nogo-66 domain of Nogo-A, myelin-associated glycoprotein, oligodendrocyte myelin glycoprotein and chondroitin sulphate proteoglycans.

**Nogo-A:** A neuronal growth inhibitory protein associated with CNS myelin.

**Nogo-66:** One of two distinct inhibitory domains of Nogo-A (residues 1026–1091 of the rat Nogo-A sequence).
